# The burden of nosocomial *staphylococcus aureus* bloodstream infection in South Korea: a prospective hospital-based nationwide study

**DOI:** 10.1186/s12879-014-0590-4

**Published:** 2014-11-14

**Authors:** Chung-Jong Kim, Hong-Bin Kim, Myoung-don Oh, Yunhee Kim, Arim Kim, Sung-Hee Oh, Kyoung-Ho Song, Eu Suk Kim, Yong Kyun Cho, Young Hwa Choi, Jinyong Park, Baek-Nam Kim, Nam-Joong Kim, Kye-Hyung Kim, Eun Jung Lee, Jae-Bum Jun, Young Keun Kim, Sung min Kiem, Hee Jung Choi, Eun Ju Choo, Kyung-mok Sohn, Shinwon Lee, Hyun Ha Chang, Ji Hwan Bang, Su Jin Lee, Jae Hoon Lee, Seong Yeon Park, Min Hyok Jeon, Na Ra Yun, The KIND Study group (Korea Infectious Diseases Study group)

**Affiliations:** National Evidence-based Healthcare Collaborating Agency, Seoul, South Korea; Seoul National University College of Medicine, Seoul, South Korea; Seoul National University Bundang Hospital, Seoul, South Korea; Seoul National University Hospital, Seoul, South Korea; Gachon University Gil Hospital, Incheon, South Korea; Ajou University College of Medicine, Suwon, South Korea; Borame Hospital, Seoul, South Korea; Inje University College of Medicine, Seoul, South Korea; Pusan National University Hospital, Busan, South Korea; Soonchunhyang University Hospital, Seoul, South Korea; Ulsan University Hospital, University of Ulsan College of Medicine, Ulsan, South Korea; Yonsei University, Wonju, South Korea; Inje University Haeundae Paik Hospital, Busan, South Korea; Ewha Woman?s University, Seoul, South Korea; Soonchunhyang University Bucheon Hospital, Bucheon, South Korea; Chungnam National University Hospital, Daejon, South Korea; Daegu Fatima Hospital, Daegu, South Korea; Kyungpook National University Hospital, Daegu, South Korea; National Medical Center, Seoul, South Korea; Pusan National University Yangsan Hospital, Yangsan, South Korea; Wonkwang University, Iksan, South Korea; Dongguk University, Goyang, South Korea; Soonchunhyang University Cheonan Hospital, Cheonan, South Korea; Chosun University, Gwangju, South Korea

**Keywords:** Staphylococcus aureus, Bacteremia, Incidence, Hospital infections

## Abstract

**Background:**

We estimated the nationwide burden of nosocomial *S. aureus* bloodstream infection (SA-BSI), a major cause of nosocomial infection, in South Korea.

**Methods:**

To evaluate the nationwide incidence of nosocomial SA-BSI, cases of SA-BSI were prospectively collected from 22 hospitals with over 500 beds over 4?months. Data on patient-days were obtained from a national health insurance database containing the claims data for all healthcare facilities in South Korea. The additional cost of SA-BSI was estimated through a matched case?control study. The economic burden was calculated from the sum of the medical costs, the costs of caregiving and loss of productivity.

**Results:**

Three hundred and thirty nine cases of nosocomial SA-BSI were included in the study: 254 cases of methicillin-resistant SA-BSI (MRSA-BSI) and 85 cases of methicillin-susceptible SA-BSI (MSSA-BSI). Death related to BSI occurred in 81 cases (31.9%) of MRSA-BSI and 12 cases (14.1%) of MSSA-BSI. The estimated incidence of nosocomial MRSA-BSI was 0.12/1,000 patient-days and that of nosocomial MSSA-BSI, 0.04/1,000 patient-days. The estimated annual cases of nosocomial BSI were 2,946 for MRSA and 986 for MSSA in South Korea. The additional economic burden per case of nosocomial SA-BSI was US $20,494 for MRSA-BSI and $6,914 for MSSA-BSI. Total additional annual cost of nosocomial SA-BSI was $67,192,559.

**Conclusion:**

In view of the burden of nosocomial SA-BSI, a national strategy for reducing nosocomial SA-BSI is urgently needed in South Korea.

**Electronic supplementary material:**

The online version of this article (doi:10.1186/s12879-014-0590-4) contains supplementary material, which is available to authorized users.

## Background

Healthcare-associated infections (HAIs) are a public health challenge, greatly affecting patient safety. The effects of HAIs include prolonged hospital stays, long-term disabilities, and unnecessary deaths [1],[2]. HAIs also impose a significant economic burden on the healthcare system. With increasing concern about patient safety in all circles of society, the prevention of HAIs has become an important aspect of the patient safety agenda [3].

Accurate determination of the burden of HAIs is fundamental in formulating HAIs management policies. In the United States, the estimated number of patients with at least 1 or more HAIs was approximately 648,000 in 2011 [4]. In the U.S., the burden of HAIs is reported periodically [4]-[6], and changing trends can be used to indicate infection control effectiveness. However, data on the nationwide burden of HAI in Asian countries are lacking. A national surveillance system, the Korean Nosocomial Infection Surveillance System (KONIS) monitors HAIs [7], but only ICU-acquired bloodstream infections (BSIs) were included. Therefore, studies estimating the burden of nationwide nosocomial infection are urgently required.

*Staphylococcus aureus* is one of the most important and devastating pathogens among HAIs, and many studies have shown that nosocomial *S. aureus* infection, especially bloodstream infection (SA-BSI) causes a tremendous burdens on the healthcare system [2],[8]-[12]. Recently, we reported the clinical characteristics and incidence of invasive *S. aureus* infection in a population-based study [13]. In the present study, we estimated the nationwide incidence and additional cost of nosocomial SA-BSI in South Korea.

## Methods

### Study population and sources of data

The population for this study consisted of all patients admitted to South Korean hospitals with over 500 beds during the June-September, 2011 study period. In 2011, there were 317 tertiary and general hospitals in South Korea, with 132,585 beds. Of these, 92 had over 500 beds, with a total number of 72,025 beds. We included 22 of a possible 92 hospitals, based on bed numbers and regional distributions (Figure?1). We considered the population of each region, and the proportion of selected hospitals in each region reflected the national proportion of the regional population. The 22 study hospitals had a total of 18,689 beds, which accounted for 25.9% of all hospitals with over 500 beds. All hospitals were acute-care hospitals.

**Figure 1:**
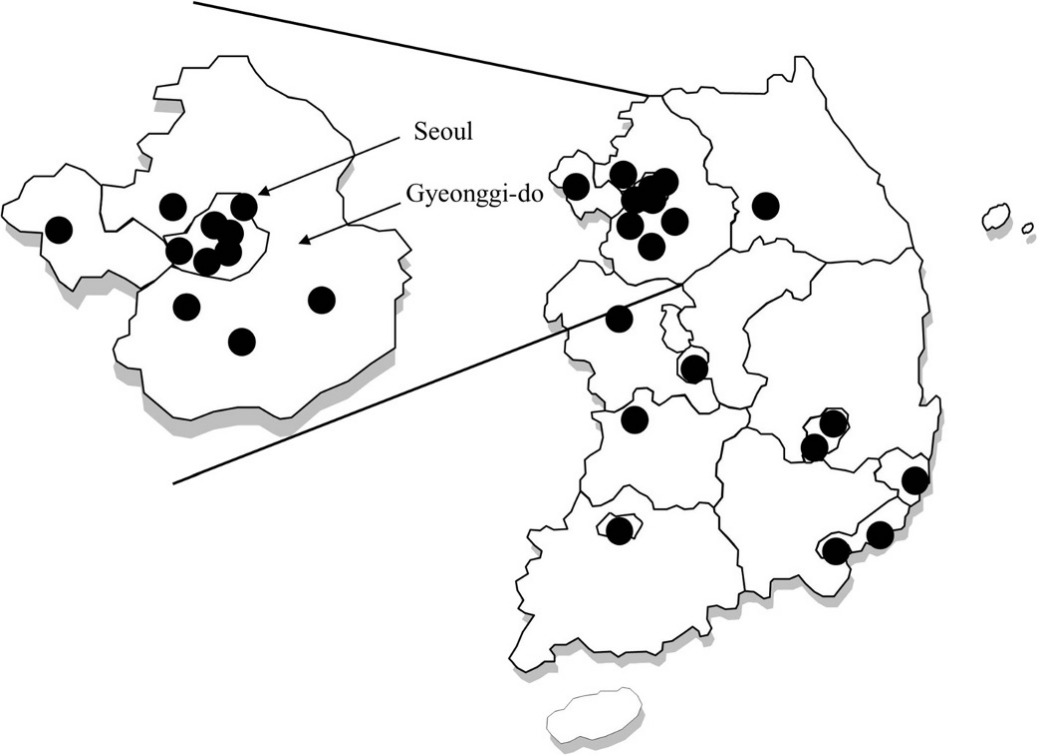
**The locations of the 22 participating hospitals.**

We obtained patient-day data for the general population and the study hospitals from the claims data of the Health Insurance Review and Assessment Service, a mandatory medical insurance system that covers the entire population of South Korea [14]. Because the study was performed in 2011 and the claims data for that year were not yet complete, the data were extrapolated using the data for the previous 5?years. Data on medical costs were obtained from the administrative database of each study hospital.

### Definitions

Nosocomial SA-BSI was defined as *S. aureus* infection which occurred after 48?hours of hospitalization, and in which *S. aureus* was isolated from blood culture. Cases of SA-BSI occurring within 48?hours of hospitalization, but for which the association between the BSI and the current hospitalization was clear were taken to be nosocomial. Exclusion criteria were as follows: 1) community-associated infection, 2) healthcare-associated community-onset infection, and 3) transfer to a study hospital after developing SA-BSI in another hospital.

Mortality associated with SA-BSI was classified as 1) definitely associated or 2) possibly associated. The former was defined as described previously [15]: 1) positive SA-BSI at the time of death or 2) death within 14?days of the documentation of SA-BSI without any other explanation. The latter was defined as: 1) death within 30?days of the documentation of SA-BSI without another explanation. Catheter-associated infection was defined according to the definition proposed by National Nosocomial Infections Surveillance System [16]; in brief, positive blood culture drawn from a central venous catheter used within 48?hours of the onset of infection, with no other apparent source of the infection identified.

Hospital charges were defined as the amount hospitals billed for healthcare services to patients. Hospital costs were defined as the sum of hospital charges and the amount that hospitals received from the Reimbursement Service, a National Health Insurance Service in South Korea. Costs were expressed in US dollars ($) at the exchange rate of 1100 Won per $ (as of 1st June, 2011).

### Matched case?control study

We conducted a matched case?control study to estimate the additional cost and length of hospitalization. In that study, nosocomial SA-BSI cases meeting the following conditions were excluded: 1) age under 18?years, 2) bloodstream infection that developed more than 30?days after admission. The latter criterion was adopted because of the difficulty of selecting control patients who were free of any nosocomial infections after 30?days of hospitalization. The control patients were selected according to the following criteria: 1) age (?5?years compared to the matched case), 2) date of admission (?2?weeks relative to the matched case), 3) confinement in the same ward as the case, and 4) underlying medical conditions similar to those of the matched case, and free of nosocomial infections. If two or more controls were identified, Charlson?s comorbidity index was used. Case?control pairs were classified into survivor and non-survivor pairs, according to whether the case patient was alive after 12?weeks of follow-up, irrespective of the outcome of the control patient.

### Data variables

Baseline demographic variables, such as age, gender, and underlying diseases were collected from the SA-BSI and control patients. Data on variables related to clinical outcomes, including length of hospitalization, mortality, and intensive care unit use were also collected.

### Data analyses

#### Estimation of annual incidence and number of SA-BSI

The annual number of SA-BSI cases was estimated in two steps. In the first step, the annual incidence of SA-BSI for the study hospitals was calculated as: (number of all nosocomial SA-BSI cases during the 4?months)???3/total patient-days of the study hospitals for the year 2011. In the second step, the annual number of SA-BSI cases was estimated as: (annual incidence of SA-BSI for the study hospitals) ? (total patient-days of the 92 hospitals for the year 2011).

#### Estimation of additional medical and societal costs of SA-BSI

We estimated the total additional costs of SA-BSI, including direct medical and societal costs. Because the additional medical cost and length of hospital stay could be influenced by survival, i.e., early deaths could lead to lower medical costs and shorter hospital stays, the calculations were performed separately for survivors and non-survivors.

The direct medical cost of SA-BSI was calculated as the product of the additional medical cost due to SA-BSI and the annual number of SA-BSI patients. The additional medical cost was calculated by subtracting the median hospital cost of the control patients from that of the corresponding SA-BSI patients. The calculation was performed separately for survivors and non-survivors.

The societal costs included the cost of caregiving, and productivity losses due to extended hospitalization and premature death. The cost of caregiving was calculated as the product of the daily cost of the hired caregiver and the excess length of stay (see above). Productivity loss due to extended hospitalization was calculated as the product of the daily wage of adults, the excess length of hospitalization, and the labor participation rate, taken as 25%. The productivity loss due to premature death was calculated from the number of deaths associated with SA-BSI and the annual wages reported by the Ministry of Labor in Korea (Labor Statistics of Korea, Ministry of Labor 2007; available from http://laborstat.molab.go.kr/). The productivity loss due to the premature death of a given patient was the sum of the annual wages up to the time that patient would have reached 65?years of age if he or she had not died. The annual discount rate was considered to be 5%. In calculating the latter, only patients under 65?years of age, the mandatory retirement age for almost all professions, were included.

Data were analyzed with Microsoft Excel 2010 (Microsoft Corp, Redmond, WA) and SPSS ver. 19.0 (SPSS Inc., Chicago, IL). The study was approved by the Institutional Review Board of the National Evidence-based healthcare Collaborating Agency and by each participating hospital. The statistical data and analyses were reviewed by KY and OSH.

## Results

### Baseline characteristics

During 4?months of the study period, 617 cases of SA-BSI were reported, of which 368 (59.6%) were cases of MRSA-BSI and 249 (40.4%) of MSSA-BSI. Community-associated infections consisted of 43 cases of MRSA-BSI and 109 cases of MSSA-BSI; 71 of the MRSA-BSI and 55 of the MSSA-BSI were healthcare-associated community-onset infections. After excluding these cases, 254 cases of MRSA-BSI and 85 cases of MSSA-BSI were included in the analysis. The baseline characteristics of the patients with nosocomial SA-BSI are shown in Table?1.

**Table 1:** **Baseline characteristics of the patients with** ***Staphylococcus aureus*** **bloodstream infections**

Characteristic	MRSA (n?=?254)	MSSA (n?=?85)	*P* -value
Gender (male/female)	167 (65.7%)/87 (34.3%)	41 (48.2%)/44 (51.8%)	0.004
Age, median (IQR)	66 (54?75)	58 (48?69)	
Location at BSI onset			
Ward	144 (56.7%)	60 (70.6%)	0.002
ER	6 (2.4%)	7 (8.2%)	
ICU	104 (40.9%)	18 (21.2%)	
Central line-associated bloodstream infection	94 (37.0%)	17 (20.0%)	0.004
Interval between admission and detection of BSI			
1-7 days	53 (20.9%)	48 (56.5%)	
8-14 days	56 (22.0%)	19 (22.4%)	
15-21 days	35 (13.8%)	6 (7.1%)	
22-30 days	19 (7.5%)	4 (4.7%)	
31-60 days	56 (22.0%)	6 (7.1%)	
61-90 days	14 (5.5%)	0	
91-180 days	16 (6.3%)	1 (1.2%)	
181-365 days	3 (1.2%)	1 (1.2%)	
Over 365?days	2 (0.8%)	0	

*Abbreviations*: *MRSA* Methicillin-resistant *S. aureus*, *MSSA* Methicillin-susceptible *S. aureus*, *IQR* Interquartile range, *BSI* Bloodstream infection, *ER* Emergency room, *ICU* Intensive care unit.

### Clinical features of nosocomial SA-BSI

Of the 254 cases of MRSA-BSI, 104 (40.9%) occurred in the ICU, compared with 18 (21.2%) of the 85 cases of MSSA-BSI. While 163 cases (64.2%) of MRSA-BSI occurred within 30?days of hospitalisation, only 77 cases (90.6%) of MSSA-BSI occurred within this time period (Table?1).

Catheters were the most frequent primary site of infection for both MRSA-BSI and MSSA-BSI. Pneumonia and surgical site infection were frequently related to MRSA-BSI, while peripheral vein phlebitis and pneumonia were frequently related to MSSA-BSI.

Deaths associated with BSI occurred in 81 cases (31.9%) of MRSA-BSI. Of these, 64 were definitely associated with BSI, and 17 were probably associated with BSI. For MSSA-BSI, 12 deaths (14.1%) were definitely associated with BSI. In a subgroup analysis of central-line associated bloodstream infections (CLABSI), 30 of 94 (31.9%) MRSA-BSI patients died, and 27 out of the 30 deaths were definitely associated with SA-BSI.

Of the 254 MRSA-BSI cases, 121 were excluded from matching with control patients for the following reasons: 91 developed BSI after more than 30?days of hospitalization, 16 were under 18?years of age (two patients were both below 18 and developed BSI after more than a month of hospitalization), and 16 could not be matched with appropriate control patients. Of the 85 MSSA-BSI cases, 28 were excluded from the control patient matching: 8 patients developed BSI after more than a month of hospitalization, 9 were under 18 (one patient was both under 18 and developed BSI after more than a month of hospitalization), and 12 patients could not be matched with appropriate controls. At the end of this matching process, 56.0% of the cases were matched with controls. Baseline characteristics, including gender, age (excluding pediatric patients), proportions of CLABSI, and mortality between included and excluded cases were similar in the MRSA and MSSA-BSI groups. The clinical and economic outcomes of the cases and controls shown in Table?2 indicate that the median medical cost of MRSA-BSI was $19,318, about 2.5 times that of controls ($7,092). For the patients with MSSA-BSI, the median medical cost was $8,030 US dollars, almost double the $4,475 US dollars for the control patients.

**Table 2:** **Clinical and economic characteristics of case and control patients with** ***S. aureus*** **bloodstream infections**

(a) MRSA vs. control			
Characteristic	MRSA (n?=?133)	Control (n?=?133)	*P* -value
Male gender	86 (64.7%)	84 (63.2%)	0.798
Age (years), median (IQR)	67 (56?76.5)	68 (56?75)	
Died	49 (36.8%)	18 (13.5%)	<0.001
Duration of hospitalization (days), mean (SD)	38.5 (26.8)	25.8 (28.0)	<0.001
Survivor pairs	44.5 (29.6)	25.2 (29.0)	<0.001
Non-survivor pairs	28.0 (16.5)	27.1 (26.2)	0.843
Charlson?s comorbidity index (SD)	4.92 (2.4)	4.5 (2.0)	0.094
Hospital cost ($), median (IQR)	19,318 (9,852-29,438)	7,092 (2,864-14,609)	
Survivor pairs	18,699 (9,660-28,369)	7,440 (3,095-14,907)	
Non-survivor pairs	19,647 (10,812-29,821)	4,875 (2,551-11,850)	
Hospital charge ($), median (IQR)	4,661 (2,240-7,959)	2,359 (870?4,128)	
Survivor pairs	4,423 (2,093-8,015)	2,394 (1,077-4,384)	
Non-survivor pairs	4,763 (2,596-7,930)	1,841 (626?3,706)	
**(b) MSSA vs. control**			
**Characteristic**	**MSSA (n?=?57)**	**Control (n?=?57)**	***P*** **-value**
Male gender	26 (45.6%)	31 (54.4%)	0.349
Age (years), median (IQR)	63 (51.5-71.5)	61 (50.5-72.0)	
Died	11 (19.3%)	4 (7.0%)	0.052
Duration of hospitalization (days), mean (SD)	29.4 (20.7)	18.1 (24.4)	0.010
Survivor pairs	31.5 (21.7)	15.5 (12.9)	<?0.001
Non-survivor pairs	17.9 (8.7)	32.8 (56.1)	0.139
Charlson?s comorbidity index (SD)	4.8 (2.4)	4.1 (2.1)	0.108
Hospital cost ($), median (IQR)	8,030 (4,998-17,733)	4,475 (2,714-9,973)	
Survivor pairs	9,247 (4,991-19,564)	4,449 (2,692-10,090)	
Non-survivor pairs	6,255 (3,952-9,815)	5,847 (2,151-10,771)	
Hospital charge ($), median (IQR)	2,054 (918?4,753)	1,517 (669?3,673)	
Survivor pairs	2,056 (845?4,822)	1,442 (619?3,309)	
Non-survivor pairs	1,713 (1,229-3,041)	1,855 (723?4,364)	

*Abbreviations*: *MRSA* Methicillin-resistant *S. aureus*, *IQR* Interquartile range, *SD* Standard deviation.

### The estimated annual incidence of nosocomial SA-BSI

The estimated annual incidence of nosocomial SA-BSI per 1,000 patient-days is shown in Table?3. The estimated annual incidence of MRSA-BSI was 0.12/1,000 patient-days, and that of MSSA-BSI was 0.04/1,000 patient-days. The estimated annual number of nosocomial SA-BSI cases was 2,946 for MRSA and 986 for MSSA. The estimated annual number of deaths was 943 for MRSA-BSI and 139 for MSSA-BSI.

**Table 3:** **Incidence of nosocomial** ***Staphylococcus aureus*** **bloodstream infection and estimated annual number of cases nationwide**

Age group		Actual number			Total patient-days for 22 hospitals			Incidence of SA BSI (per 1,000 patient-days)			Total patient-days for 92 hospitals			Estimated annual number of SA-BSI cases		
		*Male*	*Female*	*Total*	*Male*	*Female*	*Total*	*Male*	*Female*	*Total*	*Male*	*Female*	*Total*	*Male*	*Female*	*Total*
**Total**	MRSA	167	87	254	3,273,055	2,987,471	6,260,525	0.15	0.09	0.12	12,865,024	11,342,270	24,207,293	1969	991	2946
	MSSA	41	44	85				0.04	0.04	0.04				483	501	986
**0-9**	MRSA	6	7	13	389,736	292,724	682,460	0.05	0.07	0.06	1,272,094	960,391	2,232,485	59	69	128
	MSSA	3	5	8				0.02	0.05	0.04				29	49	79
**10-19**	MRSA	4	0	4	157,139	97,236	254,375	0.08	0.00	0.05	543,108	333,271	876,379	41	0	41
	MSSA	0	1	1				0.00	0.03	0.01				0	10	10
**20-29**	MRSA	2	1	3	156,072	160,269	316,341	0.04	0.02	0.03	568,594	591,027	1,159,621	22	11	33
	MSSA	0	2	2				0.00	0.04	0.02				0	22	22
**30-39**	MRSA	8	4	12	208,994	291,053	500,048	0.11	0.04	0.07	804,148	1,062,531	1,866,679	92	44	134
	MSSA	3	0	3				0.04	0.00	0.02				35	0	34
**40-49**	MRSA	9	8	17	375,999	383,098	759,097	0.07	0.06	0.07	1,501,241	1,464,574	2,965,815	108	92	199
	MSSA	4	4	8				0.03	0.03	0.03				48	46	94
**50-59**	MRSA	38	10	48	599,353	478,517	1,077,870	0.19	0.06	0.13	2,392,430	1,898,890	4,291,320	455	119	573
	MSSA	13	9	22				0.07	0.06	0.06				156	107	263
**60-69**	MRSA	48	11	59	660,593	515,558	1,176,151	0.22	0.06	0.15	2,774,688	2,005,009	4,779,697	605	128	719
	MSSA	13	9	22				0.06	0.05	0.06				164	105	268
**70-79**	MRSA	34	29	63	568,375	554,828	1,123,203	0.18	0.16	0.17	2,311,691	2,156,892	4,468,583	415	338	752
	MSSA	4	11	15				0.02	0.06	0.04				49	128	179
**>80**	MRSA	18	17	35	156,794	214,188	370,981	0.34	0.24	0.28	697,029	869,686	1,566,715	240	207	443
	MSSA	1	3	4				0.02	0.04	0.03				13	37	51

*Abbreviations*: *MRSA* Methicillin-resistant *S. aureus*, *MSSA* Methicillin-susceptible *S. aureus*, *SA-BSI S. aureus* bloodstream infection.

### The estimated additional cost of nosocomial SA-BSI

#### MRSA

The total estimated additional cost of SA-BSI is shown in Table?4. The additional medical cost for one case of MRSA-BSI was $11,259 in the survivor group and $14,772 in the non-survivor group. The costs of caregiving due to the excess days of hospitalization were $927 and $273 in the survivor and non-survivor groups, respectively. The total additional cost of MRSA-BSI was estimated to be $60,375,506 annually, corresponding to $20,494 per case of MRSA-BSI. Medical costs accounted for 60.4% of the total additional costs, and productivity loss due to premature death for 35.4%.

**Table 4:** **Estimate of the annual economic burden of nosocomial** ***S. aureus*** **bloodstream infection in South Korea**

		MRSA			MSSA		
		Additional cost per patient ($)	Annual number of patients	Additional annual cost ($)	Additional cost per patient ($)	Annual number of patients	Additional annual cost ($)
Medical cost, median							
Survivors		11,259	2003	22,551,777	4,797	847	4,063,059
Non survivors		14,772	943	13,929,996	408	139	56,712
Sub-total			2946	36,481,773		986	4,119,771
Cost of care giving						986	
Survivors		927	2003	1,857,327	818	847	693,000
Non survivors		273	943	257,182	0*	139	
Subtotal			2946	2,114,509		986	693,000
Productivity loss due to extended hospitalization							
Survivors	Male	187	1368	255,546	165	425	70,051
	Female	126	635	80,163	111	421	46,895
Non survivors	Male	55	601	33,020	0*	59	
	Female	37	342	12,698	0*	80	
Subtotal			2946	381,428		986	116,946
Productivity loss due to premature death			943	21,397,796		139	1,887,336
Total additional cost of nosocomial SA-BSI			2946	60,375,506		986	6,817,053

*Abbreviations*: *MRSA* Methicillin-resistant *S. aureus*, *MSSA* Methicillin-susceptible *S. aureus*, *SA-BSI S. aureus* bloodstream infection.

*Because hospital stays were shorter in the non-survivors in the MSSA group, no additional cost was generated.

#### MSSA

The additional medical cost of MSSA-BSI was $4,797 in the survivor group and $408 in the non-survivor group. The cost of caregiving due to excess hospitalization was $818 in the survivor group. However, in the non-survivor group, the length of hospital stay was shorter in the SA-BSI group than in the control, and no additional cost was generated. The total additional cost of MSSA-BSI was estimated to be $6,817,053 annually, which corresponds to $6,914 per case of MSSA-BSI. Medical costs accounted for 60.4% of the total additional costs, and productivity loss due to premature death for 27.7%.

## Discussion

We set out to determine the annual national financial burden of SA-BSI in South Korea. We estimated that the annual incidence of nosocomial SA-BSI was 0.16/1000 patient-days, and that there were 3,932 cases of nosocomial SA-BSI, resulting in 1,082 fatalities in 2011. We also estimated that the additional medical cost of nosocomial BSI was $12,226 per case of MRSA-BSI and $3,555 per case of MSSA-BSI. The annual economic burden of nosocomial SA-BSI in South Korea was estimated to be?>?$67.2 million, of which medical costs comprised 60.4%. This is the first nationwide nosocomial surveillance data on the clinical and financial burdens from an Asian country.

In a previous study, we reported that the estimated number of invasive *S. aureus* infection was 21,000 cases in 3 areas of South Korea, and that 55% of them were nosocomial infections [13]. This suggested that the incidence of *S. aureus* bacteremia was 43.3 per 100,000 person-years. A recent study from a single Korean hospital reported that the incidence of MRSA-BSI was 0.12/1,000 patient-days [17], which is in line with the results of the present study. The Korea Centers for Disease Control and Prevention (K-CDC) collect data on multidrug-resistant organisms via a laboratory-based surveillance system, and 4,153 cases of MRSA-BSI were reported to the K-CDC in 2012 [18], a figure in line with our findings. Unfortunately, KONIS, another surveillance system, collects data on nosocomial infections in intensive care units only, and excluding those at general wards, thus our data are not comparable with those of KONIS [7]. Notably, a Taiwanese study also found that the incidence of SA-BSI was 0.01-0.88/1,000 patient-days [19].

We included only *S. aureus* in this study, and therefore we could not estimate the incidence of nosocomial BSI of other organisms. *S. aureus* usually accounts for 10-20% of all causes of nosocomial BSI [20]-[22], and a recent study from South Korea also reported similar results [23]. If we assumed *S. aureus* comprised 10-20% of nosocomial BSI, the annual number of nosocomial BSI of all organisms could be estimated as range from 19,660 to 39,320 in 2011 in South Korea. As the total number of hospital admissions was 6,453,839 in 2011 [24], approximately 30.5-60.9 per 10,000 admitted patients are estimated to suffer from nosocomial BSI. It is of interest that the estimated burden of BSI is very close to that reported from England, which ranged from 19,202 to 28,804 patients per year [25].

Our matched-control study showed that the additional medical costs were $12,226 and $3,555, for MRSA-BSI and MSSA-BSI, respectively. These are comparable to data from Canada, with additional costs for MRSA-BSI of $6,878 to $17,553 [12],[26]. However, these costs are small compared to the additional costs in the U.S.A of $23,690 to $27,083 for MRSA-BSI and $9,661 to $19,212 for MSSA [8]-[10]. This vast difference in the additional hospital costs is mainly due to differences between the healthcare systems, especially in terms of the medical price-setting, insurance system and reimbursement policy.

Nosocomial SA-BSI imposes a large economic burden on our healthcare system. We found that the additional cost of nosocomial SA-BSI was?>?$67.2 million. This is an enormous burden, but because the proportion SA-BSI contributed to the total burden of nosocomial infections is unknown, we could not estimate the total burden of nosocomial infection. Considering that this study did not include BSI of other organisms and other HAIs such as pneumonia, urinary tract infections and wound infections, HAIs can be understood to impose a huge economic burden on our healthcare system. Studies of the nationwide economic burden of HAIs have been reported for many countries [11],[27]. De Kraker et al. estimated that the increasing burden imposed by antimicrobial-resistant organisms will surpass that of other major diseases. In the U.S.A., the total cost of the 5 major HAIs was $9.8 billion and initiatives are considered essential to reduce HAIs [28]. Our data provide an important foundation for creating a nationwide plan to reduce HAIs.

This study had certain limitations. First, the sampling period was short. We accept that a short sampling period may distort the estimates made, because of seasonal variations in disease incidence, and sampling bias. However, seasonal variations in *S. aureus* HAIs are less common than are variations in skin and soft tissue infections, community-acquired infections, and in pediatric populations [29],[30]. Many studies have found that nosocomial infection rates do not vary seasonally [31],[32]. We earlier also found that no seasonal variability was evident [13]. In surveillance of K-CDC, monthly variation in incidence of MRSA bacteremia was also not evident [18].

Secondly, because we studied only hospitals with over 500 beds, data on the nosocomial SA-BSI incidence in smaller hospitals were not included. We selected hospitals that employed infectious disease specialists, because each SAB case was required to be reviewed by such a specialist. In South Korea, more than 90% of infectious diseases specialists work in university-affiliated hospitals or large hospitals (over 500 beds), and we thus could not include medium-sized and small hospitals in our study. As it is not known whether the incidence of HAIs in small or medium-sized hospitals is higher or lower than that in large hospitals, our data cannot be extrapolated to hospitals with less than 500 beds. According to data provided by the multidrug-resistant organism surveillance system in South Korea, cases of MRSA-BSI in hospitals with 300?499 beds account for 11.7% of all cases. Therefore, considering the extent of nosocomial SA-BSI in hospitals with 500 beds or less, we estimate that more than 10-20% of all SA-BSI cases were omitted in our estimations, and, hence, the calculated cost may be at the lower end of the real nationwide figure.

## Conclusion

In conclusion, approximately 3,932 cases of nosocomial SA-BSI occurred in 2011 in the South Korean hospitals surveyed (those with 500 or more beds), and 1,082 patients died. The economic burden of nosocomial SA-BSI exceeded $67.2 million. A national strategy to reduce such infections featuring a campaign to improve hand hygiene practice, dissemination of bundle approach when managing central catheter, and periodic estimation of the burden of such infections, is urgently needed.

## Authors? contributions

CJ-K, HB-K, KH-S, ES-K, and M-O designed the study protocol; HB-K, YK-C, YH-C, JY-P, BN-K, NJ-K, KH-K, EJ-L, JB-J, YK-K, S-K, HJ-C, EJ-C, KM-S, S-L, HH-C, JH-B, SJ-L, JH-L, SY-P, MH-J and NR-Y collected the data of each participating hospital; YH-K, AR-K, SH-O performed statistical analysis and estimation; M-O managed the overall research process; CJ-K and HB-K prepared the initial draft of the manuscript; M-O revised the manuscript and finalized it; all authors were involved in manuscript review and editing. All authors read and approved the final manuscript.

## Authors’ original submitted files for images

Below are the links to the authors’ original submitted files for images. Authors’ original file for figure 1
