# Supplementary figures and images for: The burden of nosocomial staphylococcus aureus bloodstream infection in South Korea: a prospective hospital-based nationwide study
Source: BMC Infect Dis. 2014 Nov 14;14:590. doi: 10.1186/s12879-014-0590-4 (PMC4247623; doi:10.1186/s12879-014-0590-4)

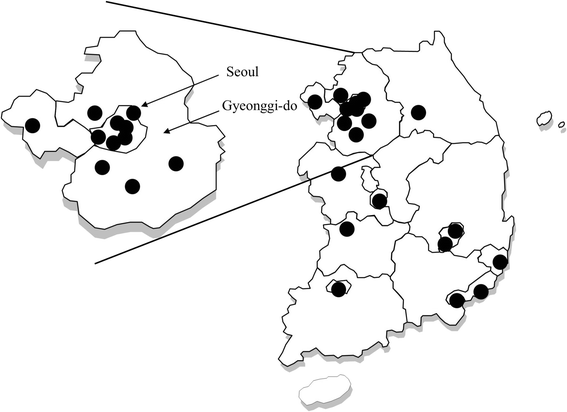

Supplement: Supplementary file 1 — Authors’ original file for figure 1 [file 12879_2014_590_MOESM1_ESM.gif]
